# Determining the Feasibility of a No‐Ultrasound Screening Tool for Early Medical Abortion in Australia

**DOI:** 10.1111/ajo.70148

**Published:** 2026-07-01

**Authors:** Catriona Melville, Philip Goldstone

**Affiliations:** ^1^ MSI Australia Melbourne Victoria Australia

**Keywords:** gestational age, induced abortion, medical abortion, mifepristone, ultrasonography

## Abstract

**Background:**

In Australia, ultrasonography to confirm gestational age (GA) and pregnancy location is a routine component of early medical abortion (EMA) care. Internationally, protocols have been developed using a history‐based screening tool to identify women not requiring ultrasound. This approach, now supported by Australian guidelines, aims to reduce unnecessary barriers to abortion care.

**Aims:**

To assess the validity and accuracy of an eligibility assessment tool for no‐ultrasound EMA care. Self‐reported estimation of GA and risk factors for ectopic pregnancy were compared to ultrasound findings.

**Materials and Methods:**

This was a multicentre prospective observational study. Patients aged 14 years and over attending for EMA completed a questionnaire, including estimation of GA using last menstrual period (LMP) or date of conception (DOC), if known, and identification of risk factors for ectopic pregnancy. All patients subsequently underwent routine abortion care, including ultrasound assessment, and outcomes were determined.

**Results:**

Of 705 patients who completed the questionnaire between 4 January 2022 and 30 August 2023, 469 were certain of their LMP or DOC (66.5%). When the screening tool was applied, 214 (30.4%) patients remained eligible for no‐ultrasound care, and all these were found to have an intrauterine pregnancy. One patient had a GA over 63 days by ultrasound.

**Conclusions:**

A history‐based screening tool determined 30% of patients eligible for no‐ultrasound EMA care. No eligible patients had an ectopic pregnancy and only one had an ultrasound confirmed GA above 63 days. Models of EMA care not requiring routine ultrasound may improve abortion access, especially for those in rural and remote areas where ultrasound availability can be challenging.

## 1 Introduction

There has been a global move to improve access to early medical abortion (EMA) by reducing the number of unnecessary and burdensome investigations required prior to the procedure [1, 2, 3, 4]. In Australia, EMA is available up to 63 days gestation using a combination of 200 mg oral mifepristone followed 36–48 h later by 800mcg buccal misoprostol [5]. Until the recent publication of an Australian national guideline [6], pre‐EMA pelvic ultrasonography has been viewed as a routine and necessary component of preabortion care to confirm the gestational age (GA) and location of the pregnancy. Ultrasound is not, however, mandated in Australian abortion legislation nor in the product information for the medical abortion composite product. The product information states that ultrasound is recommended to evaluate the duration of pregnancy and that “in the event that an ultrasound is not possible, extra caution should be exercised” [5]. Exclusion of extrauterine (ectopic) pregnancy is an additional reason routine ultrasound is utilised prior to abortion care. Approximately 11 per 1000 pregnancies are ectopic; however, the rate is reported to be lower in women seeking abortion [7]. The role of ultrasound in detecting ectopic pregnancy in asymptomatic women presenting for abortion is unclear [8].

Abortion is time‐sensitive and mandating preabortion ultrasound introduces numerous barriers to abortion access, has economic consequences for patients and services, and can cause delays in care due to inaccessibility of imaging service appointments or locations [9]. These challenges disproportionately impact those from lower socioeconomic populations, First Nations people, migrants and refugees, people without Medicare access and people living in rural and remote areas. Additionally, although some women may choose to view their ultrasound image, concerns regarding privacy and emotional distress are reported by women when accessing preabortion ultrasound [10, 11].

Internationally, EMA protocols have been developed in which ultrasound is only arranged for those who have certain features identified using a history‐based screening tool [2, 3, 4]. These “no scan” EMA protocols support the safety of first trimester medical abortion provision without routine ultrasound and are recommended in international abortion care guidelines [12, 13, 14, 15, 16, 17]. Additionally, the Royal Australian and New Zealand College of Obstetricians and Gynaecologists (RANZCOG) published the first binational evidence‐based guideline on abortion care in 2023 [6]. The guideline supports estimation of GA by methods other than ultrasonography that can be considered in abortion care for pregnancies up to 14 weeks gestation. Determination by clinical means can include last menstrual period (LMP), with or without examination. The RANZCOG guideline references but does not provide a specific eligibility assessment tool for no‐ultrasound EMA.

There is no existing Australian evidence base for use of a selective ultrasound screening tool. We therefore conducted a prospective observational study to evaluate the accuracy and safety of a history‐based screening tool in determining which patients can safely proceed with no‐ultrasound EMA care. Our primary aim was to evaluate the accuracy of the screening tool in identifying patients who were ≤ 63 days gestation and at low risk of ectopic pregnancy. A secondary aim was to assess the accuracy of self‐determined GA by certain LMP or date of conception (DOC) as a standalone parameter.

## 2 Materials and Methods

This multicentre study was conducted at six metropolitan Australian clinics within a national nongovernment sexual and reproductive health organisation, between 4 January 2022 and 30 August 2023. Clinics were located in Brisbane, the Gold Coast, Sydney, Melbourne, and Perth. A self‐completed questionnaire (File S1) was used as a screening tool to estimate GA of the pregnancy and identify risk factors for ectopic pregnancy. Normal care (including routine ultrasonography) was provided to all participants. Participants' ultrasound results and outcomes were documented following their consultation.

### 2.1 Eligibility Criteria

All patients attending a clinic for face‐to‐face EMA aged 14 years or over were invited to participate in this study. Patients who were unable to read English sufficiently or who had already undergone an ultrasound scan prior to attending the appointment were excluded from participating.

### 2.2 Statement of Ethics

This study received MSI Ethics Review Committee approval (application number 005‐23).

### 2.3 Data Collection and Statistical Analysis

Participants completed the questionnaire prior to their consultation using a QR code which generated a Microsoft Office Form. The questionnaire was completed on the patient's mobile device; however, paper copies were available for participants who did not have a mobile phone. The patient's unique medical record number was provided to them to insert at the start of the questionnaire, allowing this form to be linked to the patient's ultrasound findings. The screening tool on which the questionnaire was based was adapted for an Australian context from the Royal College of Obstetricians & Gynaecologists (RCOG) UK decision aid [18].

Provision of EMA care was not altered for study participants. All patients underwent our usual clinical process comprising consultation with a clinician and a point of care ultrasound scan to evaluate the GA and location of the pregnancy.

A paper proforma was used to document participants' ultrasound results and outcomes. This was completed by a clinician (doctor or nurse) and subsequently entered into a Microsoft Office Form. The patient completed the questionnaire, and their ultrasound findings and outcomes were linked using their unique medical record number. Accuracy of GA at clinic attendance was determined by correlation of the patient's self‐determined GA with the ultrasound scan determined GA. The patient's self‐determined GA was calculated by using the first day of their LMP or DOC if LMP was uncertain or menses were > 6 weeks apart.

Significance tests were performed with Fisher's exact test for categorical variables and Wilcoxon–Mann–Whitney test for continuous variables.

## 3 Results

Between 4 January 2022 and 30 August 2023, 750 patients who had not already had an ultrasound assessment of the current pregnancy completed the questionnaire. Of these, 45 were excluded from the study as they incorrectly entered their unique medical record number and therefore their questionnaire could not be linked to clinical findings.

The characteristics of the remaining 705 participants are summarised in Table 1. No‐ultrasound ineligible participants were more likely to be from younger age groups (15–19 and 20–24 years) compared to no‐ultrasound eligible participants. No significant difference in regional classification, income status, or employment was noted between eligible and ineligible participants.

**TABLE 1:** Characteristics of participants.

	No scan ineligible participants	No scan eligible participants	Total	*p*
	*n* = 491	*n* = 214	*n* = 705	
Age range				
15–19	46 (9%)	6 (3%)	52 (7%)	*p* < 0.001
20–24	147 (30%)	52 (24%)	199 (28%)	
25–29	129 (26%)	53 (25%)	182 (26%)	
30–34	104 (21%)	46 (22%)	150 (21%)	
35–39	46 (9%)	41 (19%)	87 (12%)	
40–49	19 (4%)	16 (7%)	35 (5%)	
Age: Median (IQR)	27 (23, 31)	29 (25, 35)	28 (23, 33)	*p* < 0.001
State of residence				
Queensland	218 (44%)	86 (40%)	304 (43%)	*p* < 0.010
New South Wales	195 (40%)	105 (49%)	300 (43%)	
Victoria	65 (13%)	14 (7%)	79 (11%)	
Western Australia	13 (3%)	9 (4%)	22 (3%)	
Regional classification of residence				
Metropolitan	463 (94%)	203 (95%)	666 (94%)	n.s
Regional	26 (5%)	11 (5%)	37 (5%)	
Remote	2 (0.4%)	—	2 (0.3%)	
Income range^†^				
Low	77 (16%)	26 (12%)	103 (15%)	n.s
Middle	411 (84%)	184 (86%)	595 (84%)	
High	3 (1%)	4 (2%)	7 (1%)	
Employment status				
Employed	358 (73%)	154 (72%)	512 (73%)	n.s
Home duties	11 (2%)	13 (6%)	24 (3%)	
Student	56 (11%)	21 (10%)	77 (11%)	
Unemployed	27 (6%)	16 (7%)	43 (6%)	
Unknown	30 (6%)	8 (4%)	38 (5%)	
Other	9 (2%)	2 (1%)	11 (1%)	
Government benefit status				
Medicare	331 (67%)	147 (69%)	478 (68%)	n.s
Medicare and healthcare card	76 (16%)	31 (15%)	107 (15%)	
No government benefits	84 (17%)	36 (17%)	120 (17%)	

*Note:* Significance tests performed with Fisher's exact for categorical variables and Wilcoxon‐Mann–Whitney test for continuous variables.

Abbreviation: n.s, not significant.

^†^ Income level is based on the 2019‐2020 Australian Tax Office annualaverage taxable income from postcodes. The income levels were split into thirdsbased on the 705 participants. Low Income: $0 ‐ $49,999, Middle Income: $50,000 ‐$117,999, High Income: $118,000+.

**FIGURE 1:**
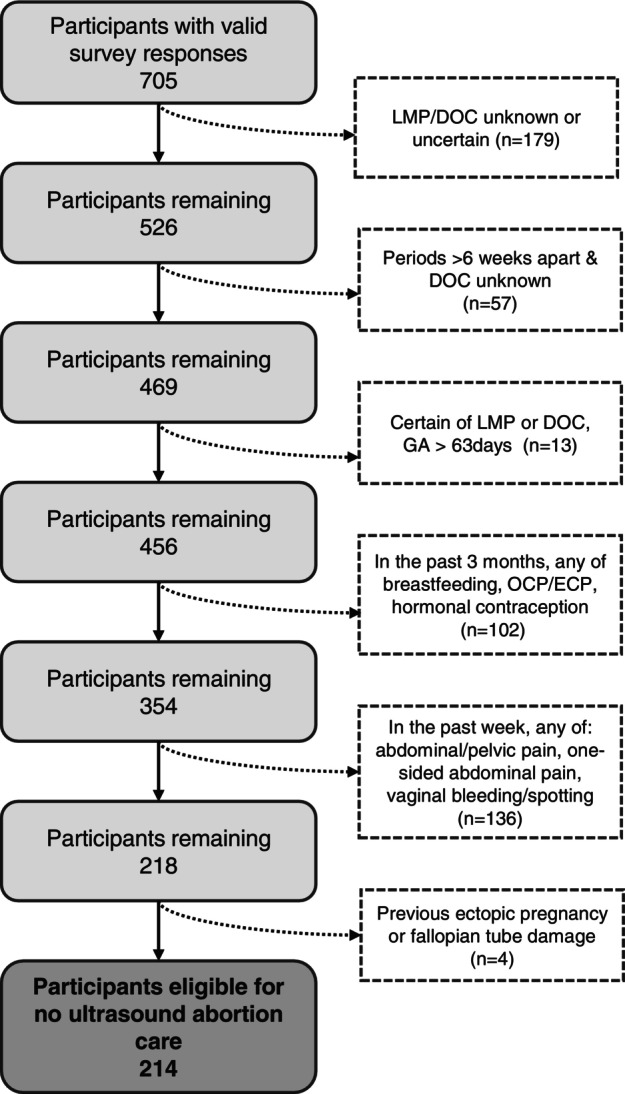
Eligibility outcomes for no scan abortion.

The questionnaire findings were used to identify which patients would have been eligible or ineligible for no‐ultrasound care. The progress of these participants through the screening tool and the questions which resulted in their exclusion are summarised in Figure 1. A total of 179 patients were uncertain of either the first day of their LMP or the DOC and were therefore immediately excluded from no‐ultrasound care. Participants reporting menstrual cycles longer than 6 weeks were also excluded (*n* = 57) unless they were certain of their DOC.

Thirteen participants had a calculated GA of > 63 days gestation using their LMP or DOC and would therefore not have been eligible for EMA based on this screening tool. 456 patients were certain of their GA by history, and this was calculated at ≤ 63 days.

Of the remaining participants, 102 were excluded due to recent breastfeeding, hormonal contraceptive, or emergency contraceptive use. A further 118 were excluded because of reported abdominal pain, 18 because of vaginal bleeding or spotting since their LMP, and four due to risk factors for ectopic pregnancy (three had a previous ectopic pregnancy and one reported previous tubal damage).

Overall, 214 of the 705 patients remained in the group eligible for no‐ultrasound care (30.4%). Following clinical assessment, only one of these participants had a GA by ultrasound of > 63 days; this pregnancy was dated at 9 weeks and 3 days (66 days) by ultrasound. A further patient was found not to be pregnant on clinical assessment including urine HCG and ultrasound scan. This patient later reported bleeding since her LMP however had denied this in the questionnaire.

One patient in the original 705 participants was found to have an ectopic pregnancy; however, application of the screening tool had excluded her from no‐ultrasound care as she reported recent hormonal contraceptive use.

To assess whether GA could be reliably determined by history alone, the accuracy of certain LMP and/or DOC as a standalone screening parameter was evaluated. Of the 526 clients who reported certainty regarding their LMP and/or DOC, 11 were excluded because their own dates determined the pregnancy to be > 63 days gestation. Of the remaining 515 participants, three had an ultrasound determined GA of > 63 days. Therefore, the accuracy of determination of GA as ≤ 63 days by certain LMP or DOC was 99.4%. All three pregnancies dated beyond 63 days on ultrasound were less than 70 days gestation, measuring 9 + 1 weeks (64 days), 9 + 3 weeks (66 days), and a twin pregnancy of 9 + 1/9 + 3 weeks (64/66 days).

## 4 Discussion

Our study found that use of a history‐based, self‐administered screening tool resulted in one third of patients being eligible for no‐ultrasound EMA care. The tool accurately identified patients who were ineligible due to GA ≥ 63 days, with > 99.5% of participants deemed eligible for no‐ultrasound care having a GA within this parameter. In other countries, EMA is available at home up to 70 days gestation using the same medication regimen available in Australia, with demonstrable safety [19]. In relation to the secondary aim of our research, none of the participants who calculated their gestation as ≤ 63 days by certain LMP or DOC were subsequently found to have a GA > 70 days. Furthermore, although ultrasound assessment in the first trimester is considered the best gestational dating method, it is accepted to have a margin of error of ±5–7 days [20]. The screening tool identified the sole participant in our study who had an ectopic pregnancy; however, she was excluded due to criteria other than typical risk factors for extrauterine pregnancy. In our cohort of 705 patients, the ectopic pregnancy incidence was 0.1%. This is consistent with the existing literature which indicates that the incidence of ectopic pregnancy among women undergoing abortion is approximately 10 times lower than in the general population [2, 21]. Patients undergoing EMA care who are found to have an ectopic pregnancy may have better outcomes likely due to enhanced surveillance resulting in earlier diagnosis and management [21, 22].

In terms of limitations, this study primarily included patients residing in metropolitan areas of Australia. It also excluded those who require support from interpreting services. It appears that younger people are less likely to be eligible for no‐ultrasound care; however, overall numbers were small. Eligibility for no‐ultrasound care in this study was likely underestimated due to the design of the screening tool. The tool was participant‐administered and applied stringent exclusion criteria, meaning that participants were automatically classified as ineligible based on specific responses. A clinician‐administered assessment would allow nuanced interpretation of symptoms such as abdominal pain or vaginal bleeding; for example, mild midline cramping in the absence of other ectopic risk factors would not necessarily preclude no‐ultrasound care. In our study, this would have enabled a further 38 patients to undergo no‐ultrasound care. Similarly, exclusion based on recent breastfeeding did not allow assessment of menstrual regularity, duration, or type of infant feeding, which would ordinarily inform clinician decision‐making. These design features likely contributed to lower observed eligibility. A recent study supports this perspective and reported that patients were more likely to self‐assess as ineligible for medical abortion care when a clinician deemed them eligible for care [23].

These findings should be interpreted alongside the international literature on no‐ultrasound abortion care. Early evidence supporting history‐based gestational assessment prior to first trimester abortion predates the widespread adoption of no‐ultrasound models of care. Foundational studies comparing LMP with ultrasound demonstrated that most individuals could report LMP with certainty, with accuracy highest earlier in gestation. A systematic review concluded that LMP based assessment is sufficiently accurate to determine eligibility for medication abortion up to 63 days' gestation, while highlighting the need for further outcome‐based research [24]. During the COVID‐19 pandemic, telemedicine models of EMA care, including no‐ultrasound, were introduced in the UK. Large cohort studies examining these new models have provided robust real‐world evidence supporting no‐ultrasound and telemedicine hybrid models of EMA. A national cohort analysis from England compared traditional in‐person ultrasound‐based care with a telemedicine hybrid model, including no‐test medical abortion up to 69 days' gestation [2]. Secondary analyses demonstrated that 61.5% of patients managed within the telemedicine hybrid pathway proceeded with no‐ultrasound telemedicine care. Importantly, rates of serious adverse events and ectopic pregnancy were not increased, and waiting times were shorter, with a greater proportion of abortions occurring at earlier gestations.

Similarly, a Scottish prospective cohort study of 663 women evaluated telemedicine abortion without routine ultrasound and demonstrated high safety, efficacy, and acceptability [25]. GA was determined using LMP alone in approximately 80% of participants, with almost all abortions occurring under 10 weeks' gestation and no cases inadvertently treated beyond 12 weeks' gestation. Rates of ectopic pregnancy were low and consistent with background population prevalence, reinforcing that routine ultrasound has limited utility as a screening test for ectopic pregnancy in asymptomatic individuals.

Together, these studies confirm that history‐based assessment reliably excludes advanced gestation and does not increase the risk of missed ectopic pregnancy when applied within structured pathways. Our findings are consistent with this literature in demonstrating accurate identification of early gestation and no increase in adverse outcomes. However, the proportion of participants deemed eligible for no‐ultrasound care in our study was lower than reported in these large UK service‐level cohorts. This likely reflects differences in study design, including the use of conservative eligibility thresholds and a participant‐administered screening tool, rather than a clinician‐led triage process. As such, our findings may represent a cautious lower‐bound estimate of eligibility within an Australian setting.

Priority areas for further research include people residing in rural and regional areas of Australia, people who are culturally and linguistically diverse and require interpreting services, and young people.

Our multicentre study determined 30% of patients eligible for no‐ultrasound EMA care using a comprehensive patient‐administered screening tool. Safety outcomes were consistent with international evidence. A clinician‐administered tool is likely to result in a higher proportion of patients being eligible for no‐ultrasound care. Mandating investigations prior to abortion care creates additional barriers to accessing a stigmatised service. The introduction of no‐ultrasound EMA care for selected individuals can support safe and timely access to this essential health service. To our knowledge, this is the first study to evaluate a defined, history‐based screening tool for this purpose in the Australian context. Our findings support implementation of Australian abortion care guidelines [6] and provide an operational screening tool appropriate for use in the local setting.

## Conflicts of Interest

The authors declare no conflicts of interest.

## Supporting information


**File S1:** Participant Questionnaire.

## Data Availability

The data that support the findings of this study are available on request from the corresponding author. The data are not publicly available due to privacy or ethical restrictions.
