# Supplementary material for: Determining the Feasibility of a No‐Ultrasound Screening Tool for Early Medical Abortion in Australia
Source: Aust N Z J Obstet Gynaecol. 2026 Jul 1;66(4):e70148. doi: 10.1111/ajo.70148 (PMC13322706; doi:10.1111/ajo.70148)
Supplement: Supplementary file 1 — File S1: Participant Questionnaire. [file AJO-66-0-s001.docx]

Appendix 1 - Participant Survey

1. Medical Record Number (MRN) (please ask reception if you are unsure)
2. What is the date today?
3. What is your age range?

16-19 20-24 25-29 30-34 35-39 40-44 45-49 50+

1. Which clinic are you visiting? (Please ask reception if you are unsure.)

[Dropdown list of clinic names]

1. Have you had an ultrasound during this pregnancy?

Yes No

1. Do you know the first day of your last menstrual period?

Yes – I’m certain Unsure – I can estimate No- I don’t know

1. What was the date your last menstrual period started (please estimate the date if you are unsure)?
2. Was it a **normal** period or was it **especially light** or **heavy**?

Normal Especially light Especially heavy Unsure

1. If especially light, what was the date of your last normal menstrual period?
2. If especially heavy, have you done a pregnancy test in the last few days?

Yes No

1. What was the result of the pregnancy test?

Positive Negative

1. Do you know the date that the pregnancy was conceived?

Yes – I’m certain Unsure – I can estimate No- I don’t know

1. What was the date that the pregnancy was conceived (estimate the date if you are unsure)?
2. Are your menstrual periods ever more than 6 weeks apart?

Yes No

1. Based on the last three months, do any of these apply to you?

I am/have been taking the contraceptive pill

I am/have been using other hormonal contraception

I have taken the emergency contraceptive pill/morning after pill

I am/have been breastfeeding

None of the above apply to me

1. Have you have experienced lower abdominal (‘tummy’) or pelvic pain more than normal menstrual period pain in the last week?

Yes No

1. Have you experienced lower abdominal (‘tummy’) pelvic pain on one side in the last week?

Yes No

1. Have you had any of the following? (Please select all that apply.)

Vaginal bleeding/ spotting within the last week

An intrauterine device (e.g. Mirena, Kyleena, copper IUD, contraceptive coil) in place currently or when you conceived

A previous ectopic pregnancy

Surgery on your fallopian tubes, including tubal ligation (sterilisation, “tubes tied”)

None of the above apply to me

1. Have you ever had an investigation or test which showed that your fallopian tubes (which connect the ovaries to the womb) are damaged?

Yes No
